# A link between gut community metabolism and pathogenesis: molecular hydrogen-stimulated glucarate catabolism aids *Salmonella* virulence

**DOI:** 10.1098/rsob.130146

**Published:** 2013-12

**Authors:** Reena Lamichhane-Khadka, Stéphane L. Benoit, Susan E. Maier, Robert J. Maier

**Affiliations:** Department of Microbiology, University of Georgia, Athens, Georgia

**Keywords:** microbial carbon utilization, carbon transport, *in vivo* pathogen growth, gut microbiome, metabolism and virulence

## Abstract

Glucarate, an oxidized product of glucose, is a major serum organic acid in humans. Still, its role as a carbon source for a pathogen colonizing hosts has not been studied. We detected high-level expression of a potential glucarate permease encoding gene *gudT* when *Salmonella enterica* serovar Typhimurium are exposed to hydrogen gas (H_2_), a gaseous by-product of gut commensal metabolism. A *gudT* strain of *Salmonella* is deficient in glucarate-dependent growth, however, it can still use other monosaccharides, such as glucose or galactose. Complementation of the *gudT* mutant with a plasmid harbouring *gudT* restored glucarate-dependent growth to wild-type (WT) levels. The *gudT* mutant exhibits attenuated virulence: the mean time of death for mice inoculated with WT strain was 2 days earlier than for mice inoculated with the *gudT* strain. At 4 days postinoculation, liver and spleen homogenates from mice inoculated with a *gudT* strain contained significantly fewer viable *Salmonella* than homogenates from animals inoculated with the parent. The parent strain grew well H_2_-dependently in a minimal medium with amino acids and glucarate provided as the sole carbon sources, whereas the *gudT* strain achieved approximately 30% of the parent strain's yield. Glucarate-mediated growth of a mutant strain unable to produce H_2_ was stimulated by H_2_ addition, presumably owing to the positive transcriptional response to H_2_. Gut microbiota-produced molecular hydrogen apparently signals *Salmonella* to catabolize an alternative carbon source available in the host. Our results link a gut microbiome-produced diffusible metabolite to augmenting bacterial pathogenesis.

## 2. Introduction

d-glucaric acid, an oxidized product of d-glucose, is a natural product found in a variety of fruits and vegetables [1,2], and in mammals [3–6]. It is also used as a dietary supplement in the form of calcium d-glucarate and it has been studied for its potential roles in reducing cholesterol levels and in cancer chemotherapy [7,8]. d-glucaric acid is normally present in the tissues and body fluids of humans [3–5] and it is a major serum organic acid in humans [9]; it approximates the blood levels of the (generally) most abundant organic acid in human serum, pyruvate. Average levels of human d-glucaric acid have been reported to be 9.2 ± 2.0 µg ml^−1^ in blood [9] and 11 to 12 µg ml^−1^ in urine [3]. The level of urine d-glucarate is used as an index of hepatic enzyme activity in humans and in experimental animals [10].

The pathogen *Salmonella enterica* serovar Typhimurium, from here on referred to as *S.* Typhimurium, are adept at surviving and growing in metabolically diverse environments both within and outside the host animal [11,12]. However, many enzymes that are synthesized and active during host infection exhibit little or no effects in virulence models [13,14]. Researchers have suggested that d-glucarate can serve as a growth substrate for a variety of microorganisms including *Escherichia coli* and related enteric bacteria [15,16]. In a study identifying some of the genes affecting glucarate utilization in *E. coli* strain K12, Roberton *et al.* [17] noted the availability of glucarate in humans at levels near the *K*_m_ of whole *E. coli* cells for the organic acid and he subsequently speculated that pathogenic *E. coli* strains may benefit by using host d-glucarate as an alternative carbon source. While the pioneering studies had indicated that d-glucarate was a potential carbon source for pathogens, its role as a carbon source for pathogenic bacteria was not studied.

From a vast compilation of data that included genome comparisons and *Salmonella* proteome expression from the pathogen in infected mice, the putative glucarate uptake and catabolism enzymes were among hundreds of enzymes that were assigned into different classes of predicted importance; some of the glucarate catabolic enzymes were expressed in the enteritis model of infection (cecum colonization), and others, but not all, were classified as ‘likely expressed’ in the typhoid fever model [13]. Still, the glucarate uptake gene *gudT* and others in the catabolic pathway were deemed to be dispensable for causing systemic salmonellosis [13]; this was defined as ‘the gene can be inactivated without significant consequences to virulence’. Also of note is that the glucarate permease and catabolism proteins were not observed among the proteome composition of log phase LB-grown *S*. Typhimurium [18]. While mutations in many individual permease genes were tested, attenuation of *Salmonella* mouse typhoid virulence via mutation of a carbohydrate permease was only observed for the mannose PTS transporter [13]; and that permease is thought to transport many different carbohydrates.

*S.* Typhimurium can colonize host (mice) organs in part by using H_2_ gas produced by the commensal microbiota; the small diffusible gas is carried in the bloodstream and can achieve levels averaging approximately 50 μM in blood-rich organs, such as liver and spleen of mice, and more than three times that level in the small intestine [19]. A gene-targeted *Salmonella* mutant strain lacking all H_2_-oxiding ability was unable to cause mouse mortality when inoculated into mice at a level whereby most of the wild-type (WT)-inoculated animals succumbed to typhoid salmonellosis within 10 days of infection [20]. One role of the Hyb enzyme was assigned via physiological studies; it augments the *Salmonella* membrane potential [21] and, as in H_2_-oxidizing *Helicobacters*, the subsequent energized membrane can facilitate carbon uptake [21,22]. Molecular hydrogen recognition via Hyb and perhaps by one of the other *Salmonella* hydrogenases stimulates expression of some *S*. Typhimurium genes associated with carbon acquisition; among the most highly up-expressed by H_2_ exposure genes was *gudT*, a gene that encodes a potential glucarate permease GudT [23]. There was 5.7-fold more (via microarray) and 10-fold more (via qRT-PCR) *gudT* expression in cells exposed to H_2_, compared with cells in the identical culture and atmospheric condition but without H_2_ [23]. Additional genes associated with glucarate catabolism, including a putative positive transcriptional regulator of glucarate catabolism genes also increased upon exposure to H_2_.

The finding that a microbiota-produced dissolved gas within hosts, namely H_2_, stimulates glucarate catabolism by *S.* Typhimurium combined with the relatively high levels of glucarate reported in animal serum/tissues led us to hypothesize that the pathogen may use H_2_ *in vivo* to express enzymes, and then drive glucarate transport. If so, glucarate catabolism would be expected to augment the bacterium's overall *in vivo* growth capacity. We thus investigated the involvement of glucarate catabolism in the virulence of *S.* Typhimurium by studying a non-polar *gudT* (permease) deletion strain. The *gudT* strain is deficient in glucarate-dependent growth compared with its parent strain and it exhibits attenuated virulence (i.e. morbidity and organ colonization). An H_2_ stimulatory effect on glucarate-dependent *Salmonella* growth was assigned by use of a mutant strain lacking all H_2_ producing ability. These results link a diffusible metabolic signal from the gut commensals to carbon transport that in turn augments pathogenicity, a link not previously documented.

## 3. Results

### 3.1. The genes and enzymes for glucarate catabolism

The genes encoding the enzymes of the d-glucarate catabolic pathway in *E. coli* have been identified and annotated [24,25]. According to the literature and available gene annotation databases, the enzymes of the d-glucarate catabolic pathways of *E. coli* and the related bacterium *S. Typhimurium* share similar functions and 97% homology (NCBI (ncbi.nlm.nih.gov), JCVI CMR (cmr.jcvi.org) BLAST tools, BioCyC (biocyc.org), EcoCyc (ecocyc.org)). d-glucarate is transported into the cells by the enzyme glucarate permease (named GudP in *E. coli* and GudT in *S*. Typhimurium) and is dehydrated to 5-dehydro-4-deoxy-d-glucarate by glucarate dehydratase, GudD [26]. d-glucarate aldolase (GarL) next reduces 5-dehydro-4-deoxy-d-glucarate into tartronate semialdehyde. Tartronate semialdehyde is further reduced to d-glycerate by tartronate semialdehyde reductase (GarR). In the final step, d-glycerate is phosphorylated to yield 2-phospho-d-glycerate by glycerate kinase (GarK). The 2-phospho-d-glycerate can be reversibly converted to 3-phospho-d-glycerate by the enzyme phosphoglyceromutase (GpmA).

### 3.2. Glucarate-dependent growth

**Figure 1.**
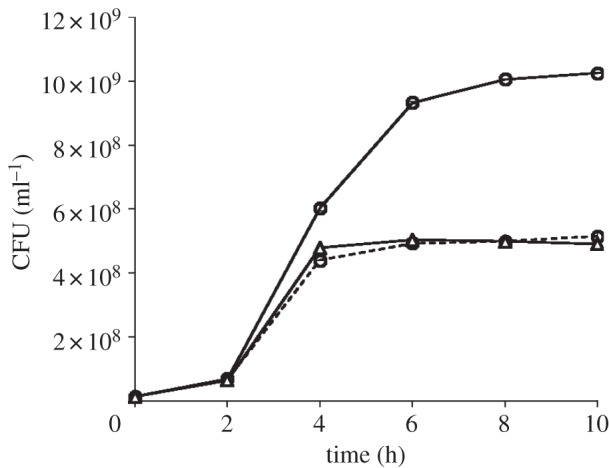
Comparison of aerobic growth of *S.* Typhimurium WT strain JSG210 (represented as circles) with RLK6/*ΔgudT* (represented as triangles) in minimal medium with 0.4% d-glucarate. Dashed line indicates WT growth without d-glucarate (control). Data points are the mean from three replicate serum bottles for each strain/condition. The standard deviation was less than 5% of the mean in every case, so that JSG210 with glucarate is greater than the lower two lines for all points at 4 h and greater at *p* ≤ 0.05 by Student's *t*-test.

*Salmonella* gene annotation sites list *gudT* as a gene-encoding putative glucarate transporter based on its high sequence similarity with probable glucarate transporters from other organisms. Its synonyms are *gudP* and *ycgZ*. In order to support a role for the putative glucarate permease, GudT, in the uptake of d-glucarate, we constructed a non-polar single gene deletion mutant of *S.* Typhimurium strain 14028 (JSG210) by using the lambda-Red system. We then compared the growth of the mutant and the parent WT strains in a minimal medium containing 0.4% d-glucarate and 0.2% casamino acids. As expected, the growth of the *gudT*-deletion strain RLK6 was significantly retarded compared with WT. The WT yielded a CFU count of approximately 1.05 × 10^9^ CFU ml^−1^ at 8 h, with a growth rate doubling time of 3 h, whereas without glucarate it yielded approximately 4 × 10^8^ CFU ml^−1^ at about 4 h after incubation and remained at that level. The pattern of RLK6 growth in glucarate-containing medium was nearly identical to that of the WT grown without glucarate (figure 1). Growth of RLK6 in glucose- or galactose-containing medium (0.4% each) was similar to WT (data not shown), suggesting that the *gudT* mutant is still able to transport and metabolize other monosaccharides. In addition, we complemented the mutant with a plasmid-containing *gudT* under the control of a (IPTG-inducible) P*_lac_* promoter. The growth of the RLK6 (pLac22-*gudT*) complemented strain in glucarate-containing medium supplemented with 5 μM IPTG was nearly identical to that of WT grown with glucarate (data not shown) confirming that *gudT* was the only gene affected in strain RLK6. Therefore, conclusions from observations on this mutant strain can be assigned to *gudT* alone. Taken together, the results indicate that GudT is important for the growth of *S.* Typhimurium on d-glucarate. In a study by Aghaie *et al.* [27], mutation of the glucarate permease gene in *Acinetobacter baylyi* ADP1 resulted in severe growth impairment on d-glucarate, albeit similar results were obtained with (*A. baylyi*) strains containing mutations in other individual genes involved in the glucarate catabolic pathway.

### 3.3. Effects of added molecular hydrogen on growth

**Figure 2.**
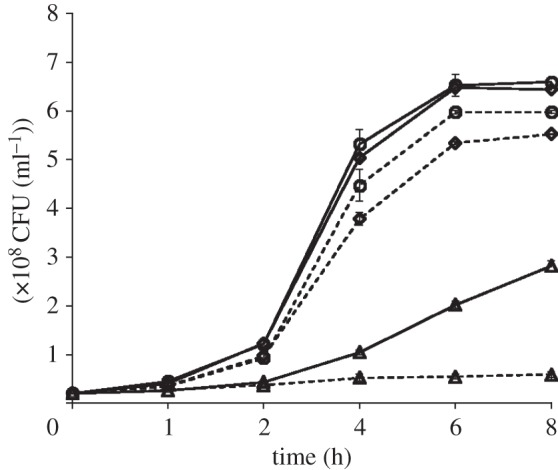
Comparison of H_2_-facilitated anaerobic growth of *S.* Typhimurium strains. Anaerobic WT/JSG210 (represented as circles), RLK7/*ΔhycC* (represented as diamonds) and RLK6/*ΔgudT* (represented as triangles) in minimal medium with 0.4% d-glucarate. Solid lines indicate growth with added H_2_ (20% v/v) and dashed lines indicate growth without added H_2_. The standard deviation was less than 4% of the mean in every case, so that the added H_2_ condition is significantly greater than without H_2_ for each individual strain for all points at 4 h and greater at *p* ≤ 0.05 by Student's *t*-test. Without H_2_, there was also a significant difference in JSG210 and the RLK7 mutant at 6 and 8 h points (*p* ≤ 0.05 by Student's *t*-test), while with H_2_, the WT was significantly greater than strain RLK6 only.

As H_2_ stimulated the expression of glucarate-use genes more than that of any other carbon acquisition genes [23], we assessed the effects of exogenously added H_2_ on the growth of *S.* Typhimurium on d-glucarate. Anaerobic respiratory growth of *S*. Typhimurium with H_2_ (reductant) and fumarate (terminal acceptor) was shown previously when amino acids were the only other carbon source provided [21]. WT and RLK6 were grown anaerobically with and without added H_2_ in a minimal medium containing 0.4% d-glucarate and 0.2% casamino acids. Addition of H_2_ enhanced the growth of both WT and RLK6, though the total CFU ml^−1^ of the mutant strain remained much lower than that of the WT in both H_2_-added and H_2_-absent conditions. Hydrogen-stimulated growth of the parent with glucarate is likely a consequence of H_2_ providing both increased glucarate catabolic gene transcription [23] and the ability of H_2_ oxidation to cause augmented proton motive force in *Salmonella* [21] that directly facilitates carbon source (e.g. glucarate) uptake. With added H_2_, RLK6 achieved about 30% of the parent strain's yield (2.0 × 10^8^ CFU ml^−1^ for RLK6; 6.5 × 10^8^ CFU ml^−1^ for WT; figure 2) and this was a statistically significant difference.

*S.* Typhimurium can use H_2_ to acquire carbon by transporting amino acids and other carbon sources into the cells from the medium [21,23]. Hence, the H_2_-augmented growth of RLK6 is likely owing to their acquisition of casamino acids or fumarate present in the medium. Theoretically, an ideal experimental condition to compare the *in vitro* growth of the strains would be to use a minimal medium containing only d-glucarate and lacking other carbons sources (e.g. casamino acids, fumarate) so that only the effects of d-glucarate could be observed. However, we could not establish such a condition, as WT grew very slowly with glucarate alone and RLK6 failed to grow at all in such a medium.

We also compared the H_2_-dependent growth of the WT with a hydrogenase mutant (RLK7) containing a deletion in the *hycC* gene that encodes the HycC subunit of the H_2_-evolving enzyme Hyc-hydrogenase [28]. This was to enable an assessment of the effects of H_2_ without interference from internally produced H_2_ from *Salmonella* metabolism. Growth experiments were performed with and without added H_2_ in minimal medium containing 0.4% d-glucarate, 0.2% casamino acids and 0.4% fumarate as described above. Sawers *et al.* [29] demonstrated that H_2_ evolution is low (between 0.016 and 0.001 µmol of H_2_ evolved per minute) when *S.* Typhimurium cells are grown under anaerobic conditions with fumarate. Added hydrogen stimulated both strains (this was seen previously with the WT, see [21]). Without added H_2_, RLK7 achieved slightly less yield than the parent, but this difference (see figure 2 legend) was significant. A statistically significant growth stimulation effect by H_2_ on RLK7 growth was observed at 4, 6 and 8 h points (e.g. for 8 h compared yield of 5.4 ± 0.2 × 10^8^ CFU ml^−1^ without H_2_ versus 6.5 ± 0.2 × 10^8^ CFU ml^−1^ with H_2_) and this is attributed to a likely positive transcriptional response of H_2_ on *gudT*, *gudD* and/or *gar* genes, but this was not examined further.

### 3.4. Mouse colonization and virulence

**Figure 3.**
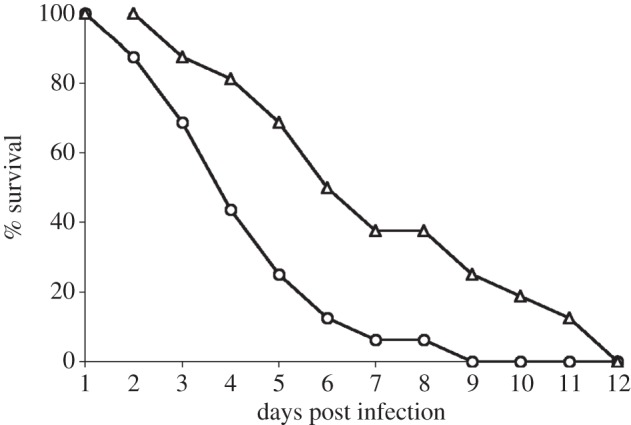
Comparison of virulence of *S.* Typhimurium strains JSG210/WT (represented as circles) and RLK6/*ΔgudT* (represented as triangles) in mice. The results shown are for 16 mice infected with each strain. The second experiment with eight mice infected with each strain showed similar results. A Wilcoxon rank-sum statistical analysis of these data was performed, testing that the distribution of dataset A (RLK6) is significantly shifted to the right of dataset B (WT), or H1: A > B using 16 data points for each strain. This test showed significance between the two groups at *p* ≤ 0.05 for a two-tailed test.

The ability of RLK6 to survive and cause infection *in vivo* was assessed by mouse stomach colonization experiments. *S.* Typhimurium is highly virulent in mice, causing typhoid fever-like disease, with rapid organ colonization and death within days of inoculation. In all mouse experiments (including the repeat experiment), the WT caused infection and death within 9 days of oral inoculation. This is consistent with the observations in our previous *S.* Typhimurium virulence study with the same WT strain [20]. The mean time of death for mice inoculated with WT was 2 days earlier than for the mice inoculated with RLK6 (figure 3). On day 9 postinoculation, 25% of the RLK6-infected mice still survived. Those remaining infected mice died, one on day 10, another on day 11 and the remaining two at day 12 postinoculation. Similar results (delayed animal mortality by the mutant compared with the parent strain) were obtained from the second experiment repeated at a later time, but using eight mice per strain (data not shown).

The organ-colonizing abilities of WT and RLK6 were compared from infected mice 4 days (96 h) after oral inoculation. The colonization numbers of bacteria in the livers and spleens were determined from four individual mice for each bacterial strain, as described previously [20]. Two mice injected with sterile PBS were included in each experiment as negative controls, and no *Salmonella* colonies were recovered from these animals. At 4 days postinoculation, liver and spleen homogenates from mice inoculated with RLK6 contained fewer viable *Salmonella* than organ homogenates from mice inoculated with WT. The organ burden numbers in mice infected with WT ranged from 1.2 × 10^6^ to 2.7 × 10^6^ (livers) and 1.5 × 10^6^ to 2.6 × 10^6^ (spleens), whereas the colonization numbers of RLK6 ranged from 0.4 × 10^6^ to 1.0 × 10^6^ (livers) and from 0.1 × 10^6^ to 1.5 × 10^6^ (spleens; table 3). The difference in the colony counts of the two strains was statistically significant at *p* < 0.05. Similar results were observed in the second colonization experiment (see table 3 legend). The results of the organ burden mouse experiments agree with the mortality results and support that the *gudT-*deleted strain RLK6 is attenuated in virulence compared with WT.

## 4. Discussion

Like many other pathogenic bacteria, *Salmonella* are catabolically flexible and robust; it is known that they survive diverse environments and can use a variety of carbon sources. From an exhaustive screen of about 600 compounds for ability to be used as possible C or N sources Gutnick *et al.* [30] found that *Salmonella* can use more than 80 different metabolites (including glucarate) as carbon sources. It is predicted that 69 of these are available in mouse tissues [14], however, mutational analysis of genes for catabolic enzymes expected to be or known to be expressed in the animal host does not often yield a disease phenotype different from the parent [13,14]. The mutant strain studied herein contained a deletion in the putative glucarate permease and other carbohydrate permeases have been studied recently. Mutation of *S*. Typhimurium genes for uptake of glucose, glucose 6-phosphate or for mannose uptake yielded little reduced capacity for *Salmonella* to replicate in epithelial colorectal adenocarcinoma cells [31]. From a broad study [13] measuring proteomic data and mouse typhoid symptoms from hundreds of *S*. Typhimurium mutant strains it was concluded that the permeases for ribose, fucose, glycerol, galactose and a number of amino acid permeases did not significantly affect virulence; only the mannose permease contributed to virulence but that permease (PTS mannose permease) also transports other carbohydrates. Similarly, from a comprehensive study in which thousands of transposon mutants could be studied via insertion-site sequencing upon animal infection, it was concluded that among transporters, only a proline-specific permease (ProY) and a peptide transporter (SapC) affected systemic typhoid virulence in mice [32]. The putative glucarate permease was not represented among their insertion mutant pool.

Following entry into the intestinal lumen, *S*. Typhimurium crosses the intestinal epithelium and is engulfed by phagocytic cells, such as dendritic cells, macrophages and granulocytes. Some of them survive this to cause severe disease. Mutational analyses by Bowden *et al.* [33] showed that the central metabolic pathway of glycolysis is required for the intracellular replication of *S.* Typhimurium within macrophages. However, mutants of *S*. Typhimurium defective in glucose uptake were still able to grow and replicate intracellularly, though at a rate lower than that of the WT [34]. While glucose may be the preferred carbon source of *S*. Typhimurium during some host–pathogen interactions [33–35], *S.* Typhimurium has enzymes to use alternative carbon sources at times when glucose is unavailable or limiting [35,36]. Considering the large number of carbon sources presumably available to *Salmonella* within the host, our result indicates that glucarate must rank among the important ones. From studying mutant strain phenotypes, proteomic, transcriptomic and other data, it was concluded [35] that *Salmonella* must sometimes rely on alternative carbon sources for systemic infections, and these may include both C_6_ and C_3_ sources. In our study, the *gudT*-deletion strain could still cause systemic disease and mortality based on the typhoid model but attenuation was evident; in light of the number of carbon sources thought to be used by *Salmonella* while residing in the host, obtaining such a phenotype is notable.

Both d-glucarate and H_2_ can be detected within animals and both substrates likely vary widely in concentration within host tissues. The glucarate catabolic genes are among a number of genes involved in carbon sequestering that are up-expressed by the pathogen in response to molecular hydrogen. This H_2_-mediated increased gene expression is most likely related to hydrogenases generating a lower potential cellular redox state, such that gene-expression-related effectors are either directly or indirectly activated. Further, some *Salmonella* appear to have more than one glucarate transporter (JCVI CMR, cmr.jcvi.org); this may mean that *Salmonella* has flexibility in acquiring the substrate at different concentrations or in different host environments. The glucarate catabolic pathway is stimulated by H_2_ [35], but we did not know whether this was relevant to colonization of the host. Molecular hydrogen is a substrate which undoubtedly is in dynamic flux within animal tissues, and it likely varies in levels, depending on the microbiome composition, host diet and proximity of the relevant tissue/organ to the source community. The gas may be a signal to *Salmonella* that it is encountering a nutritionally challenging (carbon-poor?) environment, so the pathogen can begin to catabolize an alternative carbon substrate. Still, levels of the dissolved gas measured several mm within organs (liver, spleen and heart) of live mice were well above the *K*_m_ for the reductant by membrane-bound bacterial hydrogenases [19]. Glucarate utilization must augment the survival and growth of pathogenic *Salmonella* at one or more stages of infection within the host and may also be important for colonic survival, and thus transmission. The latter environment is where the host microbial community has exhausted most of the readily usable sugars, and also where H_2_ levels are the greatest [19]; studying the role of glucarate in *Salmonella* survival there will require additional animal sampling. Nevertheless, our results link H_2_-stimulated glucarate catabolism to assisting the virulence of *S.* Typhimurium and uncover a new link between the commensal gut community and a pathogen.

## 5. Material and methods

### 5.1. Strains, growth conditions and reagents

The strains and plasmids used in this study are listed in table 1. Strains were maintained in Luria–Bertani (LB) broth or on LB Agar (LBA) plates. Appropriate antibiotics (100 µg ampicillin ml^−1^ and 25 µg kanamycin ml^−1^) were used when necessary. For growth experiments, M9 minimal medium was prepared according to the protocol provided [39] with the following modifications: NiCl_2_ (5 µM) and FeSO_4_ (5 µM) were added; d-glucarate, d-glucose or d-galactose (0.4%) was used where indicated and IPTG (5 μM) was supplemented as described. Cells were grown in serum bottles with a large headspace (15 ml culture in 165 ml bottles) at 37°C and shaking at 200 r.p.m. For aerobic growth, the bottles were maintained aseptically via cotton plugs and (loose-fit) aluminium foil. For anaerobic growth experiments, 0.4% fumarate was added to the medium, and bottles were tightly sealed with serum stoppers and aluminium crimps [28]. Anaerobic conditions with H_2_ were established by sparging the 15 ml of culture medium with inflow and outflow needles in sealed 165 ml bottles with N_2_ for 15 min, then with anaerobic mix (10% H_2_, 5% CO_2_ and 85% N_2_) for 20 min; H_2_ gas from a 100% cylinder was then injected to bring the volume of added H_2_ to 20% partial pressure [21].

**Table 1.** Strains and plasmids used in this study. FRT, flippase recombinase recognition target.

strain/plasmid	genotype/description	reference
strain		
*E. coli*		
TOP10	cloning strain	Invitrogen
*S.* Typhimurium		
JSG 210	ATCC 14028s (WT)	[28]
RLK6	JSG210*ΔgudT::*FRT (*ΔgudT*)	this study
RLK7	JSG210*ΔhycC::*FRT (*ΔhycC*)	this study
TT22971	methylating strain	John Roth
plasmids		
pCP20	Amp^r^; contains flippase gene for λ Red mutagenesis	[37]
pKD46	Amp^r^; contains λ Red genes *γ*, *β* and *exo*	[37]
pKD4	Kan^r^; contains *kan* cassette	[37]
pLac22	Amp^r^; complementation vector (*E. coli* P*_lac_*)	[38]
pLac22-gudT	pLac22 with *gudT* (*Bgl*II-*Sal*I) under P*_lac_* control	this study

### 5.2. Mutant strain construction

The deletion strain *gudT* (STM2962 single deletion) was constructed using WT strain *S.* Typhimurium ATCC 14028 (strain JSG210 or WT) and the lambda-Red system as previously described [28,37]. The *gudT*-deleted mutant was named strain RLK6, also denoted as *ΔgudT* in this paper. The deletions made using this system are non-polar and the strains do not contain antibiotic resistance markers. Additionally, a hydrogenase mutant (RLK7 or *ΔhycC*) with a single deletion of the gene *hycC* (STM2851) was constructed in a similar manner and was used to assess the effects of exogenous H_2_ in comparative growth experiments (for figure 2). The deletions were confirmed by PCR using primers complementary to the regions flanking the deleted genes and by sequencing across the deletions (Georgia Genomics Facility or GGF, University of Georgia). The plasmids and strains used in this study are listed in table 1, and the primers used are listed in table 2.

**Table 2.** Primers used in this study.

primer	primer sequence (5′ → 3′)	application
*gudT* del-F	TGAGCGTAGCTAACGCGAAATTTCAGGA- GTGCAACATGTGTAGGCTGGAGCTGCTTC	*gudT* deletion
*gudT* del-R	CCTTCATGTCCGTAATAACGGGACTGGAT- TGCGTTGTCACATATGAATATCCTCCTTA	*gudT* deletion
*gudT*-check-F	GTTTGCTTGCGTTGCCAGTA	*gudT*-deletion confirmation
*gudT*-check-R	GTTCACAGACCGGCACATTA	*gudT*-deletion confirmation
*gudT* comp-F	GTCCTAGATCTTATGAGTTCATTAAGTCAC	*gudT* complementation
*gudT* comp-R	CTGGTGTCGACTCATGATAATTGTCCTGC	*gudT* complementation
*hycC* del-F	CTTGTTTCAGCAGGCTCAGAGTGGGGA- TGCATATGTGTAGGCTGGAGCTGCTTC	* hycC* deletion
*hycC* del-R	GCGCCTGAATTAACGGATAAAACAC- ACTCATTTCATATGAATATCCTCCTTA	*hycC* deletion
*hycC*-check-F	GTGAGCTGACGTTTAATACCGA	*hycC* deletion confirmation
*hycC*-check-R	CGACCGAGCAGTTTGATAATGT	*hycC* deletion confirmation

### 5.3. Mutant complementation

Genomic DNA from WT strain JSG210 and primers *gudT* comp-F and *gudT* comp-R (table 2) were used to amplify a 1380-bp-long PCR product containing the whole *gudT* ORF as well as to introduce *Bgl*II and *Sal*I, respectively. The PCR product was gel purified, digested with both enzymes, ligated into similarly digested pLac22 vector [38] (placing *gudT* under the control of an IPTG-inducible P*_lac_* promoter) and transformed into *E. coli* TOP10. Ampicillin resistant clones were isolated and analysed and the newly generated pLac22-*gudT* plasmid was sequenced at the GGF, University of Georgia, to ensure that there was no error in the *gudT* sequence. This plasmid was first introduced in (methylating) strain TT22791 (John Roth, UC Davis) before being finally transformed into RLK6 (*gudT*) mutant. RLK6 (pLac22-*gudT*) complemented mutants were used in growth experiments in M9 supplemented with glucarate with or without IPTG (5 μM).

### 5.4. Growth curves

To confirm the role of the putative glucarate permease GudT in glucarate-dependent growth of *Salmonella* Typhimurium, the growth rates of RLK6 (*ΔgudT*) and WT strains in the minimal medium containing d-glucarate as the only carbon source were compared. Bottles (165 ml capacity) containing 15 ml minimal medium with 0.4% d-glucarate were inoculated with 1.0 × 10^7^ WT or deletion mutant *S.* Typhimurium cells. WT cells inoculated into minimal medium without d-glucarate were also included as controls. Cells were grown for up to 8 h at 37°C with shaking at 200 r.p.m. A_600_ (OD at 600 nm) was measured in 1- or 2-h intervals and cell numbers were calculated. Optical density for each time point for each strain was measured from three separate bottles, so the data point plotted is the mean ± standard deviation from three independent replicates; those points were appearing without error bars because the error bar was too small to be noted graphically. An A_600_ of 1.0 corresponds to 6.74 × 10^8^ viable *Salmonella* per ml. Standard curves of A_600_ versus CFU ml^−1^ (plate counts) confirmed that the A_600_ was proportional to viable cell numbers within the OD range used herein, including for final yield (i.e. saturation growth) numbers. The efficacy of the standard curve was separately verified for each strain. All growth rate studies were performed three times, each time with three replicate serum bottles per strain/condition with results similar to those shown (figures 1 and 2).

### 5.5. Mouse experiments

The ability of the *gudT*-deleted strain RLK6 to cause infection in mice was assessed by using the typhoid fever-mouse model [40]. Female BALB/c mice (obtained from National Cancer Institute, Frederick, MD, USA) were orally inoculated (oral gavage into the stomach) individually with cell suspensions of RLK6 and WT, following methods previously described [20]. Sixteen mice were used per strain. Cells were washed and suspended in sterile PBS and 0.1 ml volumes of the cell suspension containing 10^6^ bacterial cells were introduced orally to each mouse. The mice were observed twice daily, and immobile or nearly immobile mice were euthanized and recorded as mortality. The second experiment was performed using eight mice per strain, with results similar to those of the first experiment.

The organ bacterial burdens were determined by performing colony counts of the bacteria harvested from the livers and spleens of infected mice [20]. Four female BALB/c mice were orally inoculated with the mutant (RLK6) or the parent (WT) strains as described above. The mice were euthanized 4 days (96 h) after inoculation. Immediately after euthanizing, the livers and spleens were removed and homogenized in sterile PBS [20]. Dilutions of the homogenate were plated on Bismuth Sulfite Agar, a selective medium for *Salmonella* (Difco manual, Becton Dickinson and Co.). Colonies were counted after overnight incubation of the plates at 37°C. Two mice inoculated with sterile PBS were included as negative controls and their organs yielded no colonies. The second experiment yielded results similar to that shown in table 3.

**Table 3.** Organ colonization numbers of *S.* Typhimurium strains JSG210 (WT) and RLK6 (*gudT*-deleted mutant) in the livers and spleens of infected mice. Numbers indicate ranges of CFU per organ among four mice inoculated with each strain. Statistically significant differences between WT and RLK6 were indicated for both organs by Student's *t*-test analysis (*p* < 0.05, *n* = 4). The second experiment (also with four mice infected with each strain) showed similar results. Wilcoxon rank-sum statistical analysis using each of the latter four data points indicated differences were significant between the two strains for both organs at *p* ≤ 0.05.

strain	CFU/liver (×10^6^ cells)	CFU/spleen (×10^6^ cells)
WT	1.2–2.7	1.5–2.6
RLK6	0.4–1.0	0.1–1.5

## Notes

All animal experiments were approved by the Institutional Animal Care and Use Committee.
